# Canine paroxysmal dyskinesia—a review

**DOI:** 10.3389/fvets.2024.1441332

**Published:** 2024-07-18

**Authors:** Paul J. J. Mandigers, Koen M. Santifort, Mark Lowrie, Laurent Garosi

**Affiliations:** ^1^Department of Clinical Sciences, Expertise Centre of Genetics, Faculty of Veterinary Medicine, University of Utrecht, Utrecht, Netherlands; ^2^Evidensia Referral Hospital Arnhem, Arnhem, Netherlands; ^3^Evidensia Referral Hospital “Hart van Brabant”, Waalwijk, Netherlands; ^4^Movement Referrals: Independent Veterinary Specialists, Preston Brook, United Kingdom; ^5^Vet Oracle Teleradiology, Bedford, United Kingdom

**Keywords:** cramp, dyskinetic, hyperkinetic, movement disorder, dystonia

## Abstract

Paroxysmal dyskinesias (PDs) are a group of involuntary, hyperkinetic movement disorders that recur episodically and may last seconds to hours. An important feature of PD is that there is no loss of consciousness during the episode. Using a clinical classification, three main types of PDs have been distinguished in canine PD: (1) paroxysmal kinesigenic dyskinesia (PKD) that commences after (sudden) movements, (2) paroxysmal non-kinesigenic dyskinesia (PNKD) not associated with exercise and can occur at rest, and (3) paroxysmal exertion-induced dyskinesia (PED) associated with fatigue. Canine PDs are diagnosed based on the clinical presentation, history, and phenomenology. For the latter, a video recording of the paroxysmal event is extremely useful. An etiological classification of canine PDs includes genetic (proven and suspected), reactive (drug-induced, toxic, metabolic, and dietary), structural (neoplasia, inflammatory, and other structural causes), and unknown causes. In this review, an overview of all reported canine PDs is provided with emphasis on phenotype, genotype, and, where possible, pathophysiology and treatment for each reported canine PD.

## Introduction

Although it is likely that many movement disorders described in humans, also existed in dogs, the first report of a movement disorder in dogs dates back to 1969 (1). This is in sharp contrast with human literature. As of the 19th century, various movement disorders have been described. It is possible that Galileo Galilei was the first author who described, in the year 1,610, the existence of a physiologic tremor. Noteworthy is the publication of “shaking palsy” by Dr. James Parkinson in 1817 (2). Only when it became possible to record brief episodes of strange movements of our animals, first with video cameras and later with smartphones, both our awareness and interest in movement disorders grew, and several descriptions, of movement disorders in animals, have been published in the last decades.

The subject of this review is canine paroxysmal dyskinesia (PD). This is a group of involuntary, hyperkinetic, self-limiting, movement disorders that recur episodically and may last from seconds to hours. The other movement disorders, not subject to this review, are tremors, peripheral nerve hyperexcitability (3), and myoclonus (4).

Most PDs are the result of a dysfunction of the basal nuclei, although other central nervous system structures such as the cerebellum may also play a role (5–7). The neuroanatomy and neurophysiology of movement control were recently reviewed in the consensus statement of the International Veterinary Canine Dyskinesia Task Force (8).

PD has, in contrast with other movement disorders, a wide variety of clinical presentations (8, 9). An important feature is that during the episode, the dog remains conscious. An earlier review (9) classified canine PD according to the classification in humans (10). In analogy with human medicine, three main types of PDs have been distinguished in canine PD: (1) paroxysmal kinesigenic dyskinesia (PKD) that commences after (sudden) movements, (2) paroxysmal non-kinesigenic dyskinesia (PNKD) not associated with exercise and can occur at rest, and (3) paroxysmal exertion-induced dyskinesia (PED) associated with fatigue (9). In human medicine, the movements are described using words such as dystonia, ballism, chorea, athetosis, and choreoathetosis (10, 11). These terms cannot easily be extrapolated for use in our animals. For instance, athetosis is the involuntary contraction of (distal) limb muscles leading to a non-rhythmic bending/writhing movement of fingers, hands, or wrist (8, 10). Dogs cannot do this. Although there is great overlap in the anatomy of the central nervous system of humans and canines, there are differences that make it difficult to use the same terminology in both (5). For this reason, the recently published consensus statement was proposed to limit the terminology to be used, as there are distinct differences in anatomy and degrees of freedom of joint movement in humans and canines, preventing the use of words such as athetosis and chorea in our animals (8). Some of the used words, for instance, dystonia, have been used to describe a clinical diagnosis, whereas it is more logical to use it only as a clinical sign (12).

In this manuscript, we will use a classification based on etiology: genetic (proven and suspected), reactive (drug-induced, toxic, metabolic, and dietary), structural (neoplasia, inflammatory, and other structural causes), and unknown causes.

### Diagnosis

When confronted with a possible movement disorder, it is important to describe in detail what is seen. The International Veterinary Canine Dyskinesia Task Force recommended categorizing all movement disorders into (1) hyperkinetic versus hypokinetic, (2) paroxysmal versus persistent, and (3) exercise-induced versus not related to exercise (8). A clear description of the clinical history and phenomenology may help to differentiate a PD from focal epilepsy and/or generalized epilepsy. In 2015, the International Veterinary Epilepsy Taskforce defined epileptic seizures as *“Manifestation(s) of excessive synchronous, usually self-limiting epileptic activity of neurons in the brain. This results in transient occurrence of signs which may be characterized by short episodes with convulsions of focal motor, autonomic, or behavioral features and due to abnormal excessive of synchronous epileptic neuronal activity in the brain”* (13). If available, an electroencephalogram (EEG) reading may help to differentiate a PD from (focal) epilepsy (14). However, EEG equipment is not readily available in routine clinical practice, and therefore, it is strongly recommended to document the clinical history and phenomenology and obtain video material of what is seen, but even then, it may be difficult to draw conclusions as demonstrated in a study investigating the inter-observer agreement of paroxysmal events and epilepsy (15). Paroxysmal dyskinesias are characterized by self-limiting episodes of abnormal movement. A PD does not cause pain, the dog is conscious, and there are no autonomic signs visible. A PD can last seconds, minutes, or hours, commencing and ending abruptly. In principle, there is no post-ictal phase, and a neurological examination between episodes is normal (9). However, dogs may be exhausted after a long-lasting episode.

### Genetic PDs

#### Cavalier King Charles Spaniel/episodic falling syndrome

Episodic falling syndrome (EFS) was first described in 1983 (16). Affected dogs present themselves with progressive hypertonicity of the limbs leading to a characteristic “deer-stalking” or “praying” position. Other clinical signs may include facial muscle stiffness, stumbling, a “bunny-hopping” gait, arching of the back, and vocalization (Supplementary Video S1) (17). The episodes, triggered by exercise, stress, apprehension, or excitement (16), last from less than 1 min to several minutes. It is the first PD in which the causative mutation was identified (17). It affects young dogs (up to 4 years) and is caused by a mutation in the brevican gene (BCAN). This gene encodes a brain-specific protein of the extracellular matrix proteoglycan complex. The abnormally formed protein results in a disrupted axonal conduction/synaptic stability (17, 18). The mode of inheritance is autosomal recessive. Although all CKCS fur color variants can be crossed among each other, EFS was predominantly seen in the Ruby and Black and Tan fur variants, as the mutated allele had a higher frequency in these fur colors. Interestingly, EFS improves with age which might suggest that in time compensatory pathways may lead to a resolution of the abnormally formed protein (9, 18). For this reason, if affected CKCS only have a low frequency of these episodes, the advice could be to avoid triggers and show some hesitance in medical treatment. Affected CKCS have been treated with both clonazepam and acetazolamide (17). This PD can be classified as a PNKD.

#### Soft-Coated Wheaten Terriers

The second PD in which the causative mutation was identified occurs in the Soft-Coated Wheaten Terrier (19). The authors identified 25 affected SCWTs with episodes of rapid flexion and extension of the hind limbs with varying degrees of truncal dystonia that lasted from several minutes to several hours and could occur as often as >10/day (19). Typical episodes consisted of the flexion alternating irregularly between limbs, but sometimes both hind limbs were off the ground simultaneously (Supplementary Video S2) (19). The authors reported that in severe episodes, the front limbs were also affected (19). The episodes were not associated with exercise but could be triggered by excitement and are classified as a PNKD. A mutation in the PIGN gene (autosomal recessive) is causative for the PNKD. The PIGN gene encodes an enzyme involved in the biosynthesis of glycosylphosphatidylinositol (GPI). This protein is involved in anchoring a variety of other proteins to the cell surface. In humans, mutations in this gene are associated with multiple congenital anomalies-hypotonia-seizures syndrome-1 (MCAHS1) but not with a PD (19). Acetazolamide was tried in these dogs, and 11 dogs responded. In seven dogs, the episodes were completely abolished (20). Other treatments tried, with limited response, were clonazepam, levetiracetam, and phenobarbital (20).

#### Shetland Sheepdog

The third PD in which a genetic cause was associated with a PD was found in four female Shetland Sheepdogs. The clinical signs, mainly triggered by exercise and stress, consisted of a generalized ataxia with hypermetria and muscular hypertonia of all limbs, dystonia, normal to mildly reduced mentation, and a mild tremor (Supplementary Video S3) (21). By means of an organic acid analysis, a possible mitochondrial metabolic disease was identified. As three of the dogs were closely related (mother and two daughters), it was hypothesized to be an autosomal dominant mode of inheritance. Treatment with phenobarbital, diazepam, or levetiracetam did not resolve the clinical signs. However, a gluten-free, homemade fresh meat diet in three dogs or a tryptophan-rich, gluten-free, seafood-based diet appeared to alleviate the clinical signs (21). A genomic study revealed a case-specific missense variant in the PCK2 gene encoding the mitochondrial phosphoenolpyruvate carboxykinase 2. Sanger sequencing confirmed that all four cases carried the mutant allele in a heterozygous state (21). Several other affected female Shetland sheepdogs were identified by the first author of this manuscript (PM), and most cases responded to the dietary treatment.

#### Het Markiesje

In this small Dutch breed, several litters (approximately 10 to 12 weeks of age) were presented with clinical signs of severe PD (22). At rest, the pups were normal, but as soon as they started walking, severe tetraparesis, truncal and limb dystonia, cramping, and stumbling were observed (Supplementary Video S4). Treatment, with various medications (diazepam, phenobarbital, phenytoin, acetazolamide, and fluoxetine), was unsuccessful in all cases, and as the presentation deteriorated, elective euthanasia was performed (22). A pedigree analysis indicated an autosomal recessive mode of inheritance. Using a genome-wide association study (GWAS) and homozygosity mapping of 5 affected dogs from 3 litters, an associated locus on chromosome 31 in the region of SOD1 was identified. The DNA sequence analysis of SOD1 showed that the patients were homozygous for a frameshift mutation in the fourth codon, which means that the gene could not function. The findings were similar to a recent observation in human patients, where a loss-of-function mutation in SOD1 leads to a juvenile neurologic disease distinct from amyotrophic lateral sclerosis (22). This PD is classified as a PKD.

#### Weimaraner dogs

A recently described paroxysmal dyskinesia/dystonic ataxia was identified in Weimaraner dogs (23). The authors described four Weimaraners that were presented with episodes of an abnormal gait characterized as dystonic paroxysmal dyskinesia/ataxia. This could occasionally lead to collapse. Next to these signs, kyphosis and low head carriage were consistent features. The clinical signs were predominantly visible in the hind limbs although it could also affect the front limbs (Supplementary Video S5). The condition in these dogs deviates slightly from PD in that the dogs could have interictal signs and be abnormal for prolonged periods. Two dogs had intermittent anisocoria. This resembles episodic ataxia described in humans (24). The authors classified these episodes in the group of dystonia–ataxia syndromes (23). As written in the introduction, there are reports suggesting that in some conditions the cerebellum is involved as well (6, 7). Two cases responded well to fluoxetine treatment (23). A whole-genome sequencing revealed a private frameshift variant in the tenascin-R (TNR) gene in an affected dog leading to a non-functional protein. TNR is a member of the tenascin family of extracellular matrix glycoproteins (25). It is involved in neurite outgrowth and neural cell adhesion, proliferation and migration, axonal guidance, myelination, and synaptic plasticity (25, 26). It is exclusively expressed in the nervous system and is believed to be involved in a non-progressive neurodevelopmental disorder with spasticity and transient opisthotonos (26). The homozygotic variant was found in all four affected dogs and two unaffected heterozygotic Weimaraner dogs, but it was not found in a control group of 68 unaffected Weimaraners and 921 other dogs of various breeds (23). This PD could be classified as a PNKD.

### Presumed hereditary

#### Scottish Terrier/Scottie Cramp

Scottie Cramp is the first reported PD in dogs (1). When first reported, it was an unknown neurological disorder. After telemetered EMGs, it was concluded that it was most likely a central nervous system disorder (27). It primarily affects dogs in the first year of life (6 weeks to 18 months), and bitches are overrepresented. Clinical signs can be triggered by excitement, stress, and exercise. These include generalized cramping or hind limb hypertonicity, and skipping and may last for 5 to 20 min (Supplementary Video S6). Avoiding triggers and behavior modification helped to reduce the number of episodes (28). In time, the episodes may decrease in severity and frequency. Fluoxetine reduced the episodes in some, but not all, cases. It is presumed to be autosomal recessive, but the fact that bitches are more affected than males contradicts simple Mendelian inheritance (28). Scottie Cramp is classified as a PNKD.

#### Chinook

Familial paroxysmal dyskinesia (PNKD) was identified in 16 of 51 Chinook dogs and is characterized by an inability to stand or ambulate, head tremors, and involuntary flexion of 1 or more limbs (Supplementary Video S7) (29). The episodes varied in duration from minutes to hours, and during these periods, the dogs remained conscious. In this breed, three dogs also had concurrent generalized tonic–clonic seizures (GTCS). It is believed to be autosomal recessive (29). Whether the GTCS and PD are associated in this breed is unclear. It is possible that a genetic pleiotropy exists between PD and epilepsy as recently reported in “Het Markiesje” (22) and humans (30), but like in the Labrador Retriever, Golden Retriever, and Labradoodle, PD and GTCS can co-exist in the same breed (31, 32). Hence, it is also possible that this is just coincidental. This PD is classified as a PNKD.

#### Jack Russell Terrier

The existence of PD in the Jack Russell Terrier (JRT) is reported in a study that describes the natural history of canine paroxysmal movement disorders in Labrador Retrievers and Jack Russell Terriers (32). Over a time period of 10 years, the authors examined 23 JRTs that were referred with clinical signs of PD (Supplementary Video S8). Diagnosis was made based on the history, phenomenology, and video recordings (9, 32). The median age of onset in the JRTs was 4 years 8 months with 8 females (35%) and 15 males (65%). The episodes most commonly occurred following extremes of temperature in JRTs (32). The reported median frequency at presentation was one episode per month (range, one every 6 months to two per month). Two JRTs had cluster episodes (32). The purpose of this study was to describe the natural history if dogs did not receive treatment. During a follow-up time of over 3 years, five (22%) JRTs went into complete remission. JRTs that had a higher frequency (clusters of episodes) showed no remission. The authors concluded that treatment trials for canine PD should consider the natural history of this disease in untreated dogs before misattributing remission to specific treatment effects (32). This PD is classified as a PNKD.

#### Labrador Retriever—extreme muscular stiffness syndrome

In 2011, a novel hypertonicity syndrome in the Labrador Retriever was described (33). Although it is not a PD, it is reported in this review as next to this syndrome, PD exists in the Labrador Retriever as well (32–34). All reported cases were purebred male Labrador Retrievers. They were all young male dogs (15–47 months). The clinical signs were present for 2 to 16 months. Clinical signs consisted of continuous stiffness. It starts in the pelvic limbs and progresses to the thoracic limbs (Supplementary Video S9) (33). The dogs were treated, unsuccessfully, with various medications. Only non-steroidal anti-inflammatory drugs gave some improvement. The outcome was poor in all dogs. Four were euthanatized because of poor quality of life, two died of unrelated diseases. Only one dog remained alive, affected but stable (33). Necropsy performed in two dogs revealed astrocytosis throughout the spinal cord gray matter, reticular formation, and caudate nuclei. The disease is believed to be hereditary (X-linked).

#### Labrador Retriever—PD

The existence of PD in the Labrador Retriever is described in the already cited study that describes the natural history of canine paroxysmal movement disorders in Labrador Retrievers and Jack Russell Terriers (32). Over a time period of 10 years, the authors examined 36 Labrador Retrievers with clinical signs of PD. Diagnosis was made based on the history, phenomenology, and video recordings (9, 32). The clinical signs included dystonic hypertonia of one to all limbs, lifting one leg while lying, and inability to walk. When standing, the dogs could show hypertonia of all limbs (Supplementary Video S10). The median age of onset was 2 years and 3 months. The number of males is overrepresented [29 males (81%) and 7 females (19%)]. The episodes started after sudden movements or with excitement. The reported median frequency at presentation was once every 3 weeks. During a follow-up time of over 3 years, the frequency and duration of episodes decreased in 25 out of 36 (86%) Labradors, progressed in 5 Labradors, and remained static in 6 Labradors at follow-up (32). This PD is believed to be hereditary, and currently, a genetic study is being performed (34). This PD is classified as a PNKD.

#### Norwich Terrier

In 1984, the first report was published of an episodic muscular hypertonicity in the Norwich Terrier dog (35). In 2016, De Risio et al. (36) conducted a survey among Norwich Terrier owners. Twenty-six out of a group of 198 Norwich Terrier dogs (13%), using the questionnaire, video recordings, medical records, and telephone interviews, were classified as affected by PD (Supplementary Video S11). There was no sex predisposition. Between the episodes, all dogs were neurologically normal. The mean age at the first episode was 3 years. The episodes were characterized by sustained muscular hypertonicity in the pelvic limbs, lumbar region, and thoracic limbs, impairing posture, and locomotion without loss of consciousness (36). The episode frequency varied, and episodes lasted from 2 to 30 min. In 13 dogs, stress, anxiety, and excitement were identified as triggers. Various diets (gluten-free or meat-based) as well as different medications were tried. In none of the cases, clinical signs completely resolved with diets or medications alone (36). A pedigree analysis suggested that this PD is possibly hereditary (autosomal recessive) (36). This PD is classified as a PNKD.

#### Maltese dog

A recently performed retrospective study describes paroxysmal dyskinesia in the Maltese dog (37). The authors observed that in a group of 125 Maltese dogs, 19 had clinical signs suggestive of PD (15%). The dogs showed a combination of sudden dystonia of one or more limbs and generalized body tremors without loss of consciousness (Supplementary Video S12) (37). The episodes could occur after stress or exercise or during rest or sleep. The frequency varied from once daily to once per month or even less. The episodes lasted from 1 to 90 min with an average of 4.5 min. The mean age of clinical onset was 5.4 years. Acetazolamide was administered to six dogs, of which four responded partially. One responded to fluoxetine. Six out of seven dogs responded to a gluten-free diet. In two dogs, the PD resolved by itself. Although the authors did not perform a pedigree analysis, a hereditary cause is suspected (37). This PD is classified as a PNKD.

#### Welsh Terrier

In 2021, a case report described a levetiracetam-responsive PD in a Welsh Terrier. A 5.5-year-old, spayed, female Welsh Terrier dog exhibited, over a period of 12 months, recurrent episodes of involuntary hyperkinetic movements, abnormal muscle tone, and contractions triggered by exercise. As the PD worsened, the dog was put on levetiracetam and responded (38). Triggered by this report, another group of authors reviewed the clinical records of five referred Welsh Terriers with clinical signs of PD and performed a survey among Welsh Terrier owners. Clinical signs suggestive of PD were noted by 41 (22.8%) of 177 respondents (39). There was no sex predisposition. The median age of onset was 59 months. The episodes were characterized by sustained hypertonicity with periods of limb flexion, and abnormal head and body posture, without loss of consciousness (Supplementary Video S13) (39). The episodes varied in frequency and duration (from 30 s to 30 min). Various treatments, including gluten-free diets, with variable results, have been tried. Two additional dogs, in addition to the dog in the case report (38), responded to levetiracetam treatment (39). However, the PD was not progressive, and the frequency could decrease in time. Although no pedigree analysis was performed, a hereditary cause is suspected (39) (see Table 1).

**Table 1:** Overview of clinical characteristics of the genetic PDs.

Breed	Age	Clinical signs/classification	Triggers	Cause	Duration	Progression	Treatment	Reference(s)
Genetic								
CKCS	Young dogs	“Deer-stalking” or “praying” position. Facial muscle stiffness, stumbling, a “bunny-hopping” gait, arching of the back, or vocalization (Supplementary Video S1) PNKD	exercise, stress, excitement	Autosomal recessive. BCAN gene	< 1 min to minutes. Low frequency possible.	Improvement over time	Expectative, clonazepam, and acetazolamide	(16, 17)
SCWT	Young dogs	Rapid flexion and extension of the hind limbs with varying degrees of truncal dystonia (Supplementary Video S2) PNKD	excitement, stress	Autosomal recessive. PIGN gene.	Several minutes to several hours. Several times a day.	Continuous	Acetazolamide	(19, 20)
Shetland Sheepdog	Young dogs to any age. females	Generalized ataxia with hypermetria and muscular hypertonia of all limbs (truncal), dystonia, normal to mildly reduced mentation, and a mild tremor (Supplementary Video S3)	excitement, stress, warmer weather	Dominant. Mitochondrial PCK2 Missense Variant.	Several minutes to several hours	Progressive	A fresh meat diet with supplements	(21)
Markiesje	Pups 10–12 weeks	Generalized stiffness, varying degrees of truncal and limb dystonia, and stumbling (Supplementary Video S4) PKD	exercise, walking	Autosomal recessive. SOD1 gene.	Continuous. Only stopped at rest.	Progressive	None	(22)
Weimaraner dogs	Young dogs	Dystonic gait/ataxia. Collapse, kyphosis, low head carriage. Predominantly hind limbs, sometimes also front limbs (Supplementary Video S5) PNKD	emotional arousal or exercise	Autosomal recessive. TNR gene	5 to 15 min. Several times a day possible.	Stable	Fluoxetine	(23)

### Reactive causes

Drug-induced movement disorders are, in human medicine and most likely also veterinary medicine, a frequently observed, but not always documented, cause for myoclonus, dystonia (tardive), dyskinesias, tremors, and/or parkinsonism (40). Antiseizure medications (ASMs) and antipsychotics are the most commonly reported drugs in humans (40). In veterinary medicine, the frequently used anti-emetic metoclopramide is known to induce extrapyramidal signs such as dystonia and dyskinesia when overdosed (41). In the central nervous system, the drug antagonizes dopamine D2 receptors, which most likely explains the extrapyramidal signs (41). Other known side effects are sedation, ataxia, agitation, nausea, vomiting, and constipation. Metoclopramide leaflets for human and veterinary use warn not to use the drug in patients suffering from seizures, tardive dyskinesia, and/or dystonia (42). Another frequently used medication is metronidazole. Metronidazole can cause a cerebellar/vestibular ataxia, nystagmus, paresis, and hypermetria (43–45). Most dogs will show clear cerebellar/vestibular ataxia, but the presence of the other neurological signs may confuse a clinician. The exact mechanism is unknown. It is possible that the inhibitory neurotransmitter gamma-aminobutyric acid receptor is modulated by the drug (45). Original reports document that prolonged use at high doses increases the risk of these side effects occurring, but a recent study showed that dogs can develop the side effects at lower doses and with shorter use as well (45).

In humans, several ASMs, among others phenytoin, gabapentin, and phenobarbital, have been associated with dyskinesias (41, 46). The exact mechanism is unknown. A postulated explanation was altered neurotransmitter levels (47), although a lowered folate concentration in blood has also been associated with prolonged use of phenobarbital (48). Phenobarbital can, at a high dose, cause various side effects in dogs such as lethargy, ataxia, paresis, and recumbency (42). In 2006, phenobarbital was reported to have induced PD in an epileptic Chow Chow (47). The dog developed twitches causing an inability to stand while on phenobarbital and bromide therapy. It was diagnosed as a PD, and when the phenobarbital dose was lowered, the dog returned to normal (47). The frequently used anesthetic induction agent, propofol, can also cause clinical signs of PD. Propofol decreases the rate of dissociation of GABA from its receptors. Through prolonged binding, a chloride influx causes a hyperpolarization of the postsynaptic cell membrane (42). Propofol has been associated with PD/dystonia in humans (49, 50). Propofol is known to be able to cause myoclonus during anesthesia (Supplementary Video S14), which is thought to be caused by an imbalance of cholinergic-dopaminergic neurotransmitters (51). In 2013, a Goldendoodle showed dystonia of the neck and thoracic limbs in the recovery phase of anesthesia which included the use of propofol. As the effect of the propofol wore off, the dog became ambulatory again (51). In 2020, a crossbreed dog developed excitatory signs consisting of intermittent opisthotonic posture, limb dystonia, myoclonic jerks, paddling movements of all limbs, oculogyric movements, and excessive vocalization in the recovery phase of anesthesia induced by alfaxalone (52). This was successfully treated with the H1 antihistamine chlorphenamine (52).

The general conclusion is that signs of PD can occur if a dog receives certain medication or when it has ingested a drug or toxin that may have effects on the central nervous system. The advice is to search for known side effects and/or the mode of action. Variables such as genetic predisposition, environment, and age can play a role in the pathophysiology of these conditions and may facilitate side effects when a medication is given at a normal to higher dose (41).

### Dietary causes

The influence of nutrients or supplements as an aid in the treatment of movement disorders has been examined in several studies. Subjects of study in humans have included, among others, essential fatty acids (53), dietary protein (54), the use of a ketogenic diet (55), gluten (56–59), supplements (60), and recently the role of the microbiome (gut–brain axis) (61).

In this review, the possible effect of a gluten-free diet or other types of diets in a few breed-related PDs has been raised by the authors. The PD observed in the Shetland Sheepdog appeared to respond to a homemade fresh gluten-free meat diet (21). Some of the Maltese dogs responded favorably to a gluten-free diet (37), and the same was noted for the Welsh Terrier (39). The only breed in which it has been thoroughly examined and a clear relation established is the Border Terrier (BT).

#### Border Terrier

PD in the BT was first named “Spike’s disease” after the first dog, a Dutch BT named Spike, was diagnosed with a PD. In 2014, it was named Canine Epileptoid Cramping Syndrome (CECS) (62) although the authors at that time already classified it as a PD. In this first study, the authors described 29 young Border Terriers with clinical signs of difficulty in walking, a mild tremor, and dystonia (Supplementary Video S15). The episodes, lasting from minutes to hours, affected all four limbs as well as the head and neck in most cases. At that time, half of these dogs also developed gastro-intestinal complaints (vomiting and diarrhea), and approximately half of the dogs put on a gluten-free diet responded (62). In 2015, anti-transglutaminase 2 (TG2 IgA) and anti-gliadin (AGA IgG) were found to be elevated in 6 BTs with PD, all of which responded well to a gluten-free diet (63). Consequently, the PD was named a paroxysmal gluten-sensitive dyskinesia (9, 63, 64). In 2018, the same group measured TG2 IGA and AGA IgG in 128 BT and found that the results were conclusive to name it a gluten-sensitive PD (65). The clinical manifestation of PD in this breed has been described in 2016 in more detail (66) and attempts to unravel a genetic cause have been made in 2017 (67). The study of Stassen et al. (67) included 10 BTs in which EEG recordings were made. None of the recordings were abnormal strengthening the conclusion that it is indeed a PD (67). However, a GWAS did not identify significantly associated chromosome regions although a hereditary cause is suspected (67), but PD in the BTs is, without doubt, a gluten-sensitive PD. Gluten sensitivity, as a potential cause for neurological diseases in humans has been recognized for decades (56–59). Clinical manifestations include cerebellar ataxia (56) and PD (57), with associations postulated for conditions such as gluten encephalopathy, multiple sclerosis, peripheral neuropathies, sensorineural hearing loss, and epilepsy (68). Based on such observations and studies, it is postulated that gluten induces an immune-mediated response resulting in an antibody cross-reactivity between antigenic epitopes. In the case of gluten ataxia, this involves Purkinje cells and gluten peptides (56). In the BT, no studies, so far, have been performed investigating this possible pathogenesis in a gluten-sensitive PD (see Table 2).

**Table 2:** Overview of clinical characteristics of the presumed heritable PDs.

Breed	Age	Clinical signs/classification	Triggers	Cause	Duration	Progression	Treatment	Reference(s)
Presumed heritable								
Scottish Terrier	Young dogs. Females more affected.	Generalized cramping or hind limb hypertonicity and skipping (Supplementary Video S6)/PNKD	exercise, stress, excitement	Presumed heritable.	5 to 20 min.	Improvement over time	Fluoxetine	(27, 28)
Chinook	Young to middle aged dogs	Inability to stand or ambulate, head tremors, and involuntary flexion of 1 or more limbs (Supplementary Video S7)/PNKD	None	Autosomal recessive. Presumed heritable.	Minutes to hours	Static in time	Not reported	(29)
Jack Russell Terrier	All ages, males predominate	Dystonic hypertonia of all limbs and inability to walk or stand (Supplementary Video S8)/PNKD	Warmer weather/extremes in temperature	Unknown		Static in time	Stable, improves with age	(32)
Labrador Retriever—Stiff Muscle Syndrome	young male dogs	Continuous stiffness, first started in the pelvic limbs and progressed to the thoracic limbs. (Supplementary Video S9)	None	Hereditary, X-linked Unknown	Continuous	Deteriorate	None	(33)
Labrador Retriever—PD	Young dogs, males predominate	Dystonic hypertonia of all limbs, hypermetria, and inability to walk or stand (Supplementary Video S10)/PNKD	exercise, stress, excitement	Unknown/autosomal recessive	Several minutes to rarely hours	Static in time	Clonazepam and acetazolamide	(32)
Norwich Terrier	Average age 3 years	Sustained muscular hypertonicity pelvic limbs, lumbar region, and thoracic limbs, impairing posture, and locomotion (Supplementary Video S11)/PNKD	stress, anxiety, and excitement	Autosomal recessive	2 to 30 min	Static in time	None	(35, 36)
Maltese dog	Young dogs	Sudden dystonia of ≥1 limbs and generalized body tremors (Supplementary Video S12)/PNKD	stress, exercise but also during rest or sleep	Hereditary	1 to 90 min	Infrequently, static in time	Gluten-free diet/Acetazolamide	(37)
Welsh Terrier	Average age 5 years	Sustained hypertonicity with periods of limb flexion, abnormal head and body posture (Supplementary Video S13)/PNKD	exercise	Hereditary	30 s to 30 min	Improves in time	Levetiracetam	(38, 39)
Border Terrier	Young to average age	Sustained hypertonicity with periods of limb flexion, difficulty walking, mild tremor, abnormal head and body posture (Supplementary Video S15)/PNKD	stress, exercise	Gluten sensitivity and possible hereditary	Minutes to hours	Static in time but improves with diet change	Improves with a gluten-free diet	(61–66)

### Structural causes

Structural intra-cranial disease is also a differential diagnosis, although the exact link is unclear. It is possible that it is just a coincidental finding or that the structural cause triggers an existing PD. Earlier, we reported a secondary PNKD in a dog with multifocal forebrain lesions (9). This dog, diagnosed with meningoencephalitis of unknown origin, showed clinical signs of PD. When treated with prednisolone and cytosine arabinoside, the dog improved and showed no further signs of PD (9). A recent study investigating 100 dogs diagnosed with a head tremor demonstrated that this can be associated with an intracranial lesion in the thalamic region (69).

### Unknown causes

#### Boxer pups

In two litters, a number of pups were identified with clinical signs described at that time as episodic involuntary skeletal muscle activity similar to human paroxysmal dystonic choreoathetosis, with normal levels of consciousness. The episodes lasted from 1 to 5 min. The affected dogs were presented between 5 and 9 months of age. Males were more frequently more severely affected. Next to the dystonic limb movements, the dogs could show unilateral facial dystonia or twitching. Except for some dogs that did not improve, almost all affected dogs did improve in time. Excitement was identified as a trigger. As it only occurred in two litters and not in other pups from the same parents, a genetic cause was excluded. The hypothesis was that it was either an acquired PD or an idiopathic PD (70).

Next, to these larger descriptive studies, there are a number of more anecdotal reports: a possible paroxysmal dyskinesia in two young Dalmatian dogs (71), Bichon Frise (72, 73), German Shorthaired Pointer (Supplementary Video S16) (74), and Golden Retriever (75), and the authors of this manuscript have identified PD in several other breeds such as the Australian Labradoodle (Supplementary Video S17), Basenji (Supplementary Video S18), Chihuahua (Supplementary Video S19), cross-breeds (Supplementary Video S20) (76), Dutch Shepherd (Supplementary Video S21), Manchester Terrier (Supplementary Video S22), Pomeranian (77) (Supplementary Video S23), Pug dogs (Supplementary Video S24), Springer Spaniel (Supplementary Video S25), Dutch Stabijhoun (Supplementary Video S26), Tervueren Shepherd (Supplementary Video S27), and Yorkshire Terrier (Supplementary Video S28).

### Diagnostic approach

The first step is to document the clinical history and phenomenology and obtain video material of what is seen. A physical examination will, between the episodes of a dog with an “idiopathic” PD, be normal. If any abnormality is noted, the advice is to pursue this further. Metabolic disorders can sometimes induce clinical signs fitting with a PD (hypocalcemia) or mimic clinical signs suggestive of PD. For these reasons, a thorough hematologic and clinical chemistry blood examination is advised to exclude reactive causes such as hypocalcemia, hepato-encephalopathy, hypoglycemia, uremia, hypo and/or hypercalcemia, Addison’s disease, hypothyroidism, hyperthyroidism, and disturbances in sodium/potassium levels. Toxic causes, either acute or chronic, should always be considered as a possible cause. Acute intoxications are often accompanied by gastrointestinal and/or respiratory signs. The next step to exclude structural causes is a forebrain MRI. Although it may be tempting to skip this step in a breed in which a PD already has been described, if the possibility exists, the advice is to perform an MRI. Even in young dogs, structural and (inherited) metabolic causes and/or storage disease can occur as has been demonstrated in dogs with epilepsy (78, 79). A detailed description of the diagnosis of metabolic, toxic, and structural forebrain disorders can be found elsewhere (80).

When available, an EEG may help in the differentiation between (focal) epilepsy and PD (10, 14). If a causative mutation has been described in a dog belonging to a specific breed, such a genetic test should always be performed. As this may have implications for the population, the owner is advised to report it to the breeder/breed club.

### Treatment

When a diagnosis of PD is established, the next step is to discuss with the owner what to do. Treatment, for the published PDs, has been discussed in the previous section. Based on the published cases, it is of great importance to realize that PDs can be self-limiting in several breeds. Examples are the PDs of the CKCS (17), SCWT (20), Scottish Terrier (28), Welsh Terrier (39), JRT (32), and Labrador Retriever (32). Whether to act immediately or not has to be discussed with the owner on an individual basis. It may be prudent to first monitor the natural course of the disease and consider a period of observation of at least 2 to 3 months to assess the pattern/frequency of the episodes in that individual dog before considering a therapeutic trial, especially in breeds not reported to respond to a particular treatment or diet. If triggers are noted, it is advised to avoid these triggers whenever possible. A gluten-free diet has been tried in several breeds and appeared to have some effect in the Maltese dog (37) and Welsh Terrier (39), but its effectiveness has been proven only in the Border Terrier (63, 65). Gluten may play a role in individual cases, as a recent publication investigating the presence of TG2 IgA and gliadin IgG in various dogs with PD found elevated levels in half of the examined dogs (81). Hence, a general recommendation is to measure the presence of anti-transglutaminase and anti-deaminated gliadin and consider a gluten-free diet accordingly. The presence of such antibodies should, however, not be overinterpreted as definitive proof of a causative role of gluten in PD in every case.

As for medications used in the management of PD, the various studies cited demonstrate that there is no golden treatment. In humans, carbamazepine is the drug of choice for kinesigenic dyskinesias (10) and clonazepam for the non-kinesigenic forms (82). Acetazolamide has been used successfully in the CKCS with EFS (17) and tried with various responses in several other breeds. Anecdotal evidence includes the successful use of phenobarbital in one GSHP that responded well to this treatment (74). Interestingly, fluoxetine has been used successfully in the treatment of PD in the Weimaraner (23), but it is the observation of the authors that medical treatment often remains “trial and error”.

### Prognosis

PD may be refractory to medication but if it is an idiopathic, presumed heritable, or genetic PD, it is often non-progressive. Even “Het Markiesje” dogs did not die from the disease itself. The owners were forced to elect euthanasia as none of the dogs responded to medication and were unable to walk any distance (22). The same applies to a number of dogs described in the various cited studies. Only if the dog was refractory and its welfare and wellbeing were seriously compromised, euthanasia was chosen. The fact that some of the PDs, in time, improve even without treatment strengthens the advice to await the natural course of the PD before drastic choices are made (9, 28).

## Conclusion

Several canine PDs have been identified, and their specific features have been discussed. In five breeds up to now, the CKCS, SCTW, Shetland Sheepdog, Markiesje, and Weimaraner, a genetic cause has been identified. In several other breeds, a possible heritability has been postulated. Gluten sensitivity, as a cause, may occur in several breeds but has only been established in the Border Terrier. Diagnosing PD may be a challenge, but a logical step-wise approach is vital. Treatment is not always necessary as some PDs resolve spontaneously over time. Medical treatment results vary, and evaluation of the effect of treatment should take into account the natural course of PDs, including the possibility of spontaneous remission.

## Author contributions

PM: Conceptualization, Investigation, Writing – original draft, Writing – review & editing. KS: Writing – review & editing. ML: Supervision, Writing – review & editing. LG: Supervision, Writing – review & editing.
